# The Association between Depressive Symptoms and Physical Diseases in Switzerland: A Cross-Sectional General Population Study

**DOI:** 10.3389/fpubh.2015.00047

**Published:** 2015-03-23

**Authors:** Donja Rodic, Andrea Hans Meyer, Gunther Meinlschmidt

**Affiliations:** ^1^Division of Clinical Psychology and Epidemiology, Department of Psychology, University of Basel, Basel, Switzerland; ^2^Faculty of Medicine, Ruhr-University Bochum, Bochum, Germany

**Keywords:** comorbidity, co-occurrence, depressive symptoms, major depression, mental–physical, mental–somatic, physical disease

## Abstract

**Objective:** To estimate the association between depressive symptoms and physical diseases in Switzerland, as respective findings might inform about future estimates of mental and physical health care costs.

**Methods:** A population-based study, using data from the Swiss Health Survey collected by computer-assisted telephone interviews and additional written questionnaires during the year 2007 (*n* = 18,760) in Switzerland. The multistage stratified random sample included subjects aged 15 years and older, living in a private Swiss household with a telephone connection. Complete data were available for 14,348 subjects (51% of all subjects reached by telephone). Logistic regression analyses were used to estimate the associations between depressive symptoms and any physical disease, or a specific physical disease out of 13 non-communicable physical diseases assessed with a self-report checklist on common physical diseases. Analyses were adjusted for sex, age, education, occupation, and household income.

**Results:** In the adjusted models, depressive symptoms were associated with arthrosis and arthritis [Odds Ratio (OR) = 1.79, 95% confidence interval (CI) = 1.28-2.50] and any physical disease (OR = 1.67, 95% CI = 1.33-2.10) after controlling for multiple testing.

**Conclusion:** Our findings contribute to a better understanding of the comorbidity of depressive symptoms and arthrosis and arthritis in Switzerland and might have implications for more precise future estimates of mental and physical health care costs.

## Introduction

Depression is a global leading cause of disability (1–3), affecting 350 million people worldwide (4). According to the World Health Organization, annually more than 36 million people die of non-communicable physical diseases worldwide (5). Population-based studies around the world revealed that the presence of depressive symptoms is strongly associated with the presence of a wide range of physical diseases (6). This association has especially been reported in subjects with migraine (7, 8), asthma (7), diabetes (9, 10), chronic musculoskeletal disorders (11, 12), such as rheumatoid arthritis (13), stomach and duodenal ulcer (14), osteoporosis (15), chronic obstructive pulmonary disease (COPD) (16, 17), cardiovascular diseases (18), high blood pressure (19), myocardial infarction (20), apoplexy (21), renal disease and calculi (22), cancer (23), and allergies (24). Previous research showed that depression is associated with a wide range of physical diseases with the strongest association being reported with cardiovascular diseases (18), diabetes (9, 10), COPD (16), and chronic musculoskeletal disorders (11, 12). However, findings on the direction of the respective associations remain heterogeneous (25–28).

While there are a couple of studies, which assessed the patterns of depressive symptoms and physical diseases in large (>10,000 participants) samples (6, 7, 29–31), the majority of studies comprised much smaller samples (e.g., in the field of depression and arthrosis and arthritis 13). Therefore, high quality replication studies, assessing depression in large representative nation-wide samples are highly warranted.

While these findings cover the situation in a broad number of low-, middle-, and large high-income countries (6), less focus has been spent on small high-income countries, such as Switzerland (32). Switzerland ranks among the top three countries in overall prosperity (33) and has one of the best and most expensive health care systems worldwide (34). Therefore, it might be interesting to examine whether previous findings on the association between depression and physical diseases extend to Switzerland.

Furthermore, increased life expectancy together with an overall increased average age, not only in Switzerland (34) but also in most other regions of the world (5), is expected to result in higher prevalence of physical diseases (5, 35). At the same time, the prevalence of mental disorders within the working population is rising in Switzerland (36). Unfortunately, we are not yet in a position to estimate whether the comorbidity of chronic physical diseases and depression is about to increase as well, as the pattern of comorbidity has, to the best of our knowledge, not yet been determined in Switzerland. Our study might lay the foundation for further studies assessing the changes of mental–physical comorbidity patterns over time, as the present data derive from a nation-wide health survey conducted every 5 years in Switzerland.

Evidence suggests that patients suffering from physical diseases and comorbid mental disorders show increased mortality rates, decreased quality of life, and poorer health care outcomes as compared to patients with physical diseases without comorbid mental disorders (37, 38). Thus, scrutinizing the associations between mental disorders and physical diseases could provide implications for improved health care for patients with comorbid mental and physical conditions.

The presence of depressive symptoms in subjects with a physical disease increases treatment costs for this disease, as compared to subjects without depressive symptoms suffering from the same disease (39–41). To date, a potential effect of depression on treatment costs for related physical diseases was not taken into account for precise estimates of the economic burden caused by depression in Switzerland (42). Thus, it is essential to estimate the prevalence of conditions comorbid with depression, and this article might therefore contribute to more precise future estimates of costs related to depression in Switzerland and comparable countries.

Additionally, to estimate the costs for co-occurring depressive symptoms and physical diseases, it is important to not simply add the separate costs related to depressive symptoms (costs related to depression per person, multiplied by number of subjects with depression) to the separate costs related to physical diseases (costs related to physical disease, multiplied by number of subjects with physical disease), which is referred to as double counting (1). Thus, by accounting for co-occurrence of these conditions, double counting can be avoided. Thereby, the estimation of the prevalence of comorbid conditions might contribute to more precise future estimates of costs of co-occurring depressive symptoms and physical diseases.

The aim of this article was to estimate the association between depressive symptoms and physical disease in Switzerland.

## Materials and Methods

### Design and sample

Data were drawn from the Swiss Health Survey (SHS) conducted by the Swiss Federal Statistical Office and carried out by the M.I.S.-Trend S.A Institute in Lausanne and Gümligen in 2007 (43). The data collection and storage for the SHS do not require formal approval by an ethical committee, as this data collection, preparation, and storage are specifically permitted under Swiss law and participants could decline to participate or withdraw at any time (SR 431.012.1; SR 431.112.1). The authors of this manuscript were not involved in data collection. Anonymized data for further analysis were obtained upon signing a data confidentiality and privacy contract from the Swiss Federal Statistical Office upon request.

**Figure 1:**
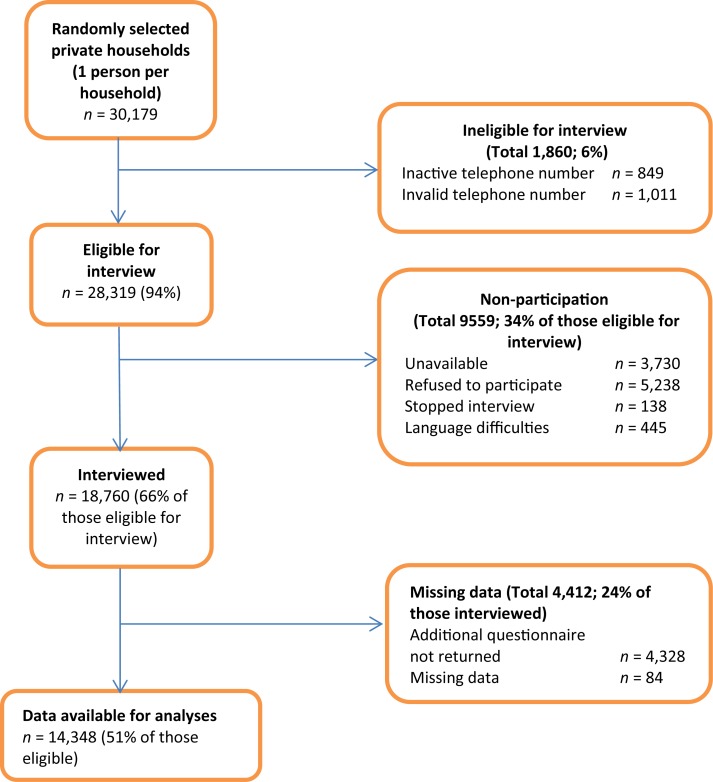
**Flowchart of study participants**.

The SHS is a periodic, nation-wide, and cross-sectional survey consisting of a computer assisted telephone interview followed by a written questionnaire. The multistage stratified random sample included subjects aged 15 years and older, living in a private Swiss household with a telephone connection in 2007. Out of the 30,179 randomly selected private households a total of 18,760 subjects completed the telephone interview, corresponding to a response rate of 66% (Figure 1). The additional written questionnaire was completed by 77% (*n* = 14,348) of the interview participants (Figure 1).

The SHS, among others, provides representative data on mental and physical health and healthcare utilization (43). A detailed description of the SHS methodology and sampling has been described elsewhere (44) and descriptive results of the SHS have been reported in the Swiss Health Observatory of 2013 (45).

### Assessment of depressive symptoms

Depressive symptoms were assessed with the screening scale for depression (46) from the World Health Organization Composite International Diagnostic Interview Short Form (CIDI-SF) in the SHS. Participants were asked whether they felt sad, blue, or depressed for two or more weeks during the past 12 months, followed by a series of questions on symptoms related to depression, such as weight gain or loss. A summary score was calculated based on the positive responses to these symptom-related questions (range 0–7). Subjects scoring above a cut-off of three symptoms were screened positive for major depression. A detailed description of the CIDI-SF scoring has been reported elsewhere (47). The CIDI-SF has demonstrated good performance for depression in large community samples (48–50). Moreover, the CIDI-SF screening scale for depression demonstrated good reliability and validity in the SHS sample (51).

### Assessment of physical diseases

In the SHS, common non-communicable physical diseases were assessed with a self-report checklist, based on the European Health Interview Survey (52). Participants were asked whether they received recent (more than 12 months ago) or current (during the past 12 months) treatment by a medical doctor for each physical disease out of a list of 13 conditions. These conditions included migraine, asthma, diabetes, arthrosis and arthritis, stomach ulcer and duodenal ulcer, osteoporosis, COPD and emphysema, high blood pressure, myocardial infarction, apoplexy, renal disease and renal calculi, cancer and blastoma, and allergies like hay fever. Comparable checklists have shown high accord with medical records (53, 54) and better accuracy than consultation rates in national studies (55).

### Assessment of socio-demographic measures

We considered sex, age, education, occupation, and equivalized household income as covariates as these characteristics have previously been linked to depression (56–58) and physical diseases (59). Age was treated as categorical variable grouped into six categories: 15–30, 31–40, 41–50, 51–60, 61–70, and 71 years and above. The educational level comprised three categories: mandatory school, secondary school, and university (60). Occupation was categorized into the four categories middle/higher senior management, office employees/other non-manual professions, small business owners/independent craftspeople, and workpeople, based on the Erikson–Goldthrope–Portocarero classification of occupation (60, 61). The equivalized household income, a continuous index representing a gradient between low and high income, was calculated by dividing the monthly household income by the weighted number of person in the household, where a weight of 1.0 was set for the first person over 15 years, 0.5 for each additional person, and 0.3 for each child under 15 years (60, 62).

### Statistical analyses

The goal was to assess the relationship between depressive symptoms and various types of physical disease. We run 14 separate logistic regression models (unadjusted models) with depressive symptoms (yes/no) as predictor, and each of the 13 physical diseases (yes/no) or all combined diseases (“any physical disease”; yes/no) as outcomes. We additionally ran the same 14 models, controlling for the following covariates: sex, age, education, occupation, and equivalized household income (adjusted models). Results reported in the main test derived from the adjusted models. Results from the unadjusted models can be found in the online Supplementary Material (Table S1). A significance test for the predictor depressive symptoms was obtained by comparing the full model with the same model while excluding the predictor depressive symptoms, using a likelihood ratio test.

All analyses were carried out with the recommended standardized sampling weights for the SHS sample (44). Descriptive analyses were performed using the IBM Statistical Package for Social Sciences 21 (IBM Corp. Released 2012, IBM SPSS Statistics for Windows, Version 21.0, Armonk, NY: IBM Corp.). Regression analyses were performed using R, version 2.14.2 (63), including the R package survey (64). All tests were two-tailed with a significance level of 0.05. We controlled for multiple testing using the Holm–Bonferroni method (65).

## Results

Complete data were available for 14,348 subjects (51% of all subjects reached by telephone). Table 1 presents the characteristics of this subsample. Of the 14,348 subjects, 740 (weighted percentage = 5.2) were screened positive for depressive symptoms. The number of subjects with specific physical diseases, stratified by the presence/absence of depressive symptoms, is presented in Table 1.

**Table 1:** **Sample characteristics (*n* = 14,348)**.

Socio-demographics	*N*	% (%^w^)
Sex
Female	8,057	56.2 (51.1)
Male	6,291	43.8 (48.9)
Age in years
15–30	2,221	15.5 (24.0)
31–40	2,589	18.0 (17.5)
41–50	2,714	18.9 (19.5)
51–60	2,354	16.4 (15.1)
61–70	2,382	16.6 (12.5)
71+	2,088	14.6 (11.3)
Educational level
Mandatory school	1,725	12.0 (11.7)
Secondary level	8,660	60.4 (61.7)
University	3,963	27.6 (26.7)
Occupation
Workpeople	3,428	23.9 (25.4)
Small business owners/ independent craftspeople	1,164	8.1 (7.6)
Office employee/other non-manual professions	3,497	24.4 (22.9)
Middle/higher senior management	5,628	39.2 (38.3)
Unknown	631	4.4 (5.8)

	**Median (25th–75th percentile)**	
Equivalized household income in CHF^a^	3,700 (2,500–5,333)	

	***n* (%^w^)**	
**Physical diseases**	**With depressive symptoms *n* = 740**	**Without depressive symptoms *n* = 13,608**

Any physical disease	270 (35.8)	4, 127 (30.4)
Migraine	46 (6.1)	315 (2.3)
Asthma	28 (3.7)	354 (2.6)
Diabetes	23 (3.1)	398 (2.9)
Arthrosis, arthritis	82 (10.9)	1, 104 (8.1)
Stomach ulcer, duodenal ulcer	16 (2.1)	124 (0.9)
Osteoporosis	30 (4.0)	327 (2.4)
COPD, emphysema	23 (3.1)	249 (1.8)
High blood pressure	80 (10.6)	1, 841 (13.5)
Myocardial infarction	8 (1.1)	173 (1.3)
Apoplexy	4 (0.5)	73 (0.5)
Renal disease, renal calculi	17 (2.3)	125 (0.9)
Cancer, blastoma	28 (3.7)	216 (1.6)
Allergies, hay fever	67 (8.9)	806 (5.9)

**n*, unweighted number of subjects; %^w^, weighted percentage; CHF, Swiss Franc, COPD, chronic obstructive pulmonary disease*.

*^a^Gradient between low and high income*.

The results of the logistic regression models are presented in Table 2. Depressive symptoms were associated with an increased risk for migraine, diabetes, arthrosis and arthritis, COPD and emphysema, renal disease and renal calculi, cancer and blastoma, allergies like hay fever, and the risk for any physical disease. However, after Holm–Bonferroni correction, only the association of depressive symptoms with arthrosis and arthritis remained (OR = 1.79, 95% CI = 1.28-2.50).

**Table 2:** **Adjusted logistic regression models^a^ of physical diseases predicted by depressive symptoms (*n* = 14,348)**.

Physical disease	*n* of subjects with condition	OR (95% CI)	*p*_uncontrolled/_*p*_controlled_^b^
Any physical disease	4,397	1.67 (1.33,2.10)	<0.001*/–
Migraine	361	2.07 (1.27,3.39)	0.008*/0.087
Asthma	382	1.49 (0.91,2.44)	0.137/0.694
Diabetes	421	2.05 (1.17,3.61)	0.023*/0.182
Arthrosis, arthritis	1,186	1.79 (1.28,2.50)	0.002*/0.021*
Stomach ulcer, duodenal ulcer	140	1.44 (0.71,2.94)	0.340/1.000
Osteoporosis	357	1.86 (0.91,3.81)	0.116/0.694
COPD, emphysema	272	2.33 (1.29,4.21)	0.012*/0.123
High blood pressure	1,921	1.29 (0.92,1.81)	0.159/0.694
Myocardial infarction	181	0.90 (0.33,2.45)	0.828/1.000
Apoplexy	77	1.37 (0.33,5.83)	0.680/1.000
Renal disease, renal calculi	142	2.63 (1.31,5.29)	0.017*/0.149
Cancer, blastoma	244	2.45 (1.40,4.30)	0.005*/0.064
Allergies, hay fever	873	1.59 (1.05,2.41)	0.042*/0.297

*OR, odds ratio; CI, confidence interval; COPD, chronic obstructive pulmonary disease*.

*p < 0.05.

*^a^Results are adjusted for age, sex, education, occupation, and household income*.

*^b^Controlled for multiple testing using Holm–Bonferroni method*.

## Discussion

The aim of the present analyses was to estimate the association between depressive symptoms and physical diseases in a representative population sample in Switzerland. The presence of depressive symptoms was associated with an increased risk for any physical disease and after correcting for multiple testing with an increased risk for arthrosis and arthritis.

To the best of our knowledge, this is the first study to determine the pattern of comorbidity of depressive symptoms and physical diseases in Switzerland. Our results are in line with previous studies reporting associations between depression and physical diseases in general (66–70) and associations between depression and arthrosis and arthritis (6, 11, 71, 72) in particular. Moreover, our findings are in line with findings on the high prevalence of depression in patients with different physical diseases (26), as well as in patients with musculoskeletal disorders, such as osteoarthritis and rheumatoid arthritis (11, 12). In addition, a recent meta-analysis confirmed the high prevalence of depression in patients with rheumatoid arthritis (13). However, our findings are not in line with previous studies reporting comorbid depressive symptoms in patients with cardiovascular disease (18, 73), diabetes (9, 10), or COPD (16, 17). However, this might be due to a relatively lower statistical power and related less precise estimation of comorbidity for those physical diseases under study with lower prevalence (including, stomach and duodenal ulcer, COPD and emphysema, myocardial infarction, apoplexy, renal disease and calculi, cancer, and blastoma).

Due to the cross-sectional nature of this study, we are not able to draw conclusions about the causality of the estimated associations. We consider three different models to explain the estimated associations: (A) depressive symptoms may lead to physical diseases, more specifically to arthrosis and arthritis; (B) physical diseases, among other arthrosis and arthritis, may lead to depressive symptoms; (C) there may be common factors leading to both depressive symptoms and physical diseases, especially arthrosis and arthritis.

With regard to (A), it has been shown that depressive symptoms cause disability (1) and are related to a loss of interest in everyday activities (74). Moreover, depressive symptoms have been associated with unfavorable health behavior, such as smoking (75, 76). Therefore, one may speculate that depressive symptoms may, via unfavorable health behavior, pave the way for chronic physical disease. More specifically, depression is related to reduced physical activity (77) and that has been associated with musculoskeletal disorders, such as osteoarthritis (12).

With regard to (B), one may speculate that having a chronic physical disease decreases quality of life (78, 79), which might affect the mental health status of a person with a physical disease (23, 80, 81) resulting in reduced subjective well-being and ability to cope with everyday life in individuals with poor mental health (82). The perception of being unable to cope with everyday requirements has been associated with depression (83, 84) and this might lead to a loss of interest in daily activities, resulting in decreased positive reinforcement to participate in everyday life, which has been associated with depression (85, 86). More specifically, arthrosis and arthritis have been associated with reduced physical activity (87) and it has been shown that physical activity has a beneficial effect on depressive symptoms (88–90). Thus, arthrosis and arthritis might therefore favor the development of depressive symptoms.

With regard to (C), we can only speculate about potential further factors. Interestingly, different immune parameters, such as cytokines, have been associated with both depressive symptoms (91) and physical diseases (68, 92). Whether these parameters represent mediators of the observed associations or common risk factors for depression and physical diseases is largely unknown. Thus, further research is needed to scrutinize the mechanisms underlying the association between depression and physical diseases.

Our analysis has several strengths: first, we included a representative population sample totaling 14,348 subjects. Second, the presence of physical diseases was verified by receiving treatment from a medical doctor. Third, we controlled for potential confounders that have been previously linked to depression (56–58) and physical diseases (59). Finally, we corrected for multiple testing, as recommended (93–95).

This study also has limitations and our findings need replication in an independent dataset. First, the non-response rate within the SHS might have caused a selection bias (96). Therefore future studies aiming to a higher response rate are needed in Switzerland. Second, we were not able to determine the severity of depressive symptoms and any of the physical diseases, as this information was not available from the SHS data. This might have led to underestimation of conditions present in the general population. Therefore, studies precisely aiming to investigate the prevalence and severity of depressive symptoms and depressive disorders, as well as physical diseases, in the general population are urgently needed in Switzerland. Future studies should allow for the detection of subtle variations of depressive symptoms in the general population, and estimate how these variations relate to comorbidity with physical diseases. Third, physical diseases were assessed with a self-report checklist. However, comparable checklists highly accorded with medical records (53, 54) and showed better accuracy than consultation rates in national studies (55). Furthermore, all data on physical diseases and corresponding disease categories were predefined in the SHS. Thus, we were not able to analyze the conditions arthrosis and arthritis as separate diagnoses, as this information was not available from the SHS data. However, both conditions are diseases of the musculoskeletal system and connective tissue and involve similar symptoms, such as pain and inflammation (97).

With regard to generalizability of the findings, caution is warranted. It is questionable whether our findings on the association between depressive symptoms and physical diseases in Switzerland are applicable to other high-income countries because of Switzerland’s high performing health care system (34). However, our results on the association between depressive symptoms and arthrosis and arthritis in Switzerland do not substantially differ from representative community-based results in Canada (7), which is also a high-income country (32).

Due to the cross-sectional nature of our study, we abstained from expressing a clear preference for one of the possible explanations given for the associations between depressive symptoms and any physical diseases, respectively, arthrosis and arthritis. In this study, depressive symptoms were associated with any physical disease and depressive symptoms were related to an increased risk for arthrosis and arthritis. Our findings may contribute to a better understanding of the comorbidity of depressive symptoms and physical diseases, as well as provide the basis for a better understanding of the changes of mental–physical comorbidity over time in Switzerland. We found that depressive symptoms were related to arthrosis and arthritis, which might have implications for clinical practice. They suggest considering to encourage the screening of subjects with arthrosis and arthritis for depressive symptoms and vice versa. Early recognition of the co-occurrence of depressive symptoms with arthrosis or arthritis might allow for an improved coordination of interdisciplinary treatment (specialized therapies depending on the outcome of the screening) and this may positively influence the course of both conditions. Treatment costs of physical diseases are higher for subjects with co-occurring depressive symptoms than for subjects without depressive symptoms suffering from the same disease (39–41). Thus, knowledge about the comorbidity of depression and physical diseases may allow a more comprehensive estimate of the overall costs related to depression. Therefore, our results might have implications for more precise future estimates of costs related to depression, including secondary costs, related to increased expenditures for co-occurring physical conditions in Switzerland. Additionally, by accounting for co-occurrence of comorbid conditions, double counting can be avoided (1), which might contribute to more precise future estimates for costs of co-occurring depressive symptoms and physical diseases.

Future studies should focus on the mechanisms underlying the association between depressive symptoms and the increased risk for physical disease in general and arthrosis and arthritis in particular.

## Author Contributions

DR drafted the manuscript. DR and GM conceived and designed the research question and analyses. AM performed the statistical analyses. DR and GM interpreted the results. GM supervised the study and revised the manuscript for important intellectual content. All authors read and approved the final manuscript.

## Conflict of Interest Statement

The authors, Donja Rodic, Andrea Hans Meyer, and Gunther Meinlschmidt, declare that they have no conflict of interest. The authors alone are responsible for the content and writing of the paper. Gunther Meinlschmidt receives funding from the Swiss National Science Foundation (project no. 100014_135328) and from the Korea Research Foundation within the Global Research Network Program (project no. 2013S1A2A2035364). The funders had no role in study design, data collection and analysis, decision to publish, or preparation of the manuscript.

## Supplementary Material

The Supplementary Material for this article can be found online at http://www.frontiersin.org/Journal/10.3389/fpubh.2015.00047/abstract

## Abbreviations

CI, confidence interval; CIDI-SF, Composite International Diagnostic Interview Short Form; COPD, chronic obstructive pulmonary disease; OR, odds ratio; SHS, Swiss health survey.
