# Supplementary material for: The Association between Depressive Symptoms and Physical Diseases in Switzerland: A Cross-Sectional General Population Study
Source: Front Public Health. 2015 Mar 23;3:47. doi: 10.3389/fpubh.2015.00047 (PMC4370044; doi:10.3389/fpubh.2015.00047)
Supplement: Supplementary file 1 [file Table_1.DOCX]

***Supplementary Material***

**The association of depressive symptoms and physical diseases in Switzerland: a cross-sectional general population study**

**Donja Rodic_1_^1^, Andrea H. Meyer_2_^1^, Gunther Meinlschmidt_3_^1,2^***

^1^Department of Psychology, Division of Clinical Psychology and Epidemiology, University of Basel, Basel, Switzerland ^2^Faculty of Medicine, Ruhr-University Bochum, Bochum, Germany

*** Correspondence:** Gunther Meinlschmidt, Department of Psychology, University of Basel, Missionsstrasse 62a, 4055 Basel, Switzerland.

gunther.meinlschmidt@unibas.ch

1. **Supplementary Table**

We run 14 separate logistic regression models (unadjusted models) with depressive symptoms (yes/no) as predictor, and each of the 13 physical diseases (yes/no) or all combined diseases (“any physical disease”; yes/no) as outcomes. Results from the unadjusted models are presented in Table S1.

| **TABLE S1. Unadjusted Logistic Regression Models of Physical Diseases predicted by Depressive Symptoms (*n* = 14,348)** | | | | | |
| --- | --- | --- | --- | --- | --- |
|  | Physical Disease |  | *n* of subjects with condition | OR [95% Cl] | p_uncontrolled/_p_controlled_^b^ |
|  | Migraine |  | 364 | 2.57 [1.62, 4.09] | 0.000*/0.005* |
|  | Asthma |  | 384 | 1.38 [0.86, 2.22] | 0.208/0.832 |
|  | Diabetes |  | 423 | 1.25 [0.72, 2.16] | 0.449/0.897 |
|  | Arthrosis, Arthritis |  | 1,191 | 1.44 [1.04, 1.99] | 0.039*/0.349 |
|  | Stomach Ulcer, Duodenal Ulcer |  | 141 | 1.53 [0.82, 2.86] | 0.209/0.832 |
|  | Osteoporosis |  | 360 | 1.90 [1.01, 3.56] | 0.068/0.534 |
|  | COPD, Emphysema |  | 274 | 1.69 [0.98, 2.91] | 0.080/0.559 |
|  | High Blood Pressure |  | 1,934 | 0.79 [0.57, 1.08] | 0.130/0.770 |
|  | Myocardial Infarction |  | 183 | 0.53 [0.22,1.30] | 0.128/0.770 |
|  | Apoplexy |  | 78 | 1.02 [0.30, 3.41] | 0.973/0.973 |
|  | Renal Disease, Renal Calculi |  | 142 | 2.51 [1.33, 4.74] | 0.012*/0.132 |
|  | Cancer, Blastoma |  | 245 | 2.12 [1.25, 3.57] | 0.011*/0.132 |
|  | Allergies, Hay Fever |  | 880 | 1.58 [1.06, 2.34] | 0.032*/0.324 |
| Any Physical Disease | |  | 4,421 | 1.35 [1.08, 1.68] | 0.010*/− |
| OR = Odds Ratio, Cl = Confidence Interval  COPD = Chronic obstructive pulmonary disease  * p < 0.05  ^b^ Controlled for multiple testing using Holm-Bonferroni method | | | | | |
